# The study of CaO and MgO heterogenic nano-catalyst coupling on transesterification reaction efficacy in the production of biodiesel from recycled cooking oil

**DOI:** 10.1186/s40201-015-0226-7

**Published:** 2015-10-23

**Authors:** Kambiz Tahvildari, Yasaman Naghavi Anaraki, Reza Fazaeli, Sogol Mirpanji, Elham Delrish

**Affiliations:** Department of chemistry, Islamic Azad University, North Tehran Branch, Tehran, Iran; Modeling and Optimization Research Center in Science and Engineering, Islamic Azad University, South Tehran Branch, Tehran, Iran; Nano-ophthalmology Department, Stem Cells Preparation Unit, Eye Research Center, Farabi Eye Hospital, Tehran University of Medical Sciences, Tehran, Iran

**Keywords:** Biodiesel production, Heterogenic Nano-catalyst, Recycled cooking oil, Transesterification reaction

## Abstract

**Background:**

Fossil fuels’ pollution and their non-renewability have motivated the search for alternative fuels. Some common example of seed oils are sunflower oil, date seed oil, soy bean oil. For instance, soy methyl and soy-based biodiesel are the main biodiesel. Biodiesel is a clean diesel fuel that can be produced through transesterification reaction. Recycled cooking oil, on the other hand, is one of the inexpensive, easily available sources for producing biodiesel.

**Results:**

This article is aimed at production of biodiesel via trans-esterification method, Nano CaO synthesis using sol-gel method, and Nano MgO synthesis using sol-gel self-combustion. Two catalysts’ combination affecting the reaction’s efficacy was also discussed. Optimum conditions for the reaction in the presence of Nano CaO are 1.5 % weight fracture, 1:7 alcohol to oil proportion and 6 h in which biodiesel and glycerin (the byproduct) are produced. Moreover, the optimum conditions for this reaction in the presence of Nano CaO and Nano MgO mixture are 3 % weight fracture (0.7 g of Nano CaO and 0.5 g of Nano MgO), 1:7 alcohols to oil proportion and 6 h.

**Conclusions:**

Nano MgO is not capable of catalyzing the transesterification by itself, because it has a much weaker basic affinity but when used with Nano CaO due to its surface structure, the basic properties increase and it becomes a proper base for the catalyst so that CaO contact surface increases and transesterification reaction yield significantly increases as well. This study investigates the repeatability of transesterification reaction in the presence of these Nano catalysts as well.

## Introduction

Today, the growing need for new energy sources in transportation is one of the primary challenges for different countries. Scientists are compelled to find alternatives to currently used fuels because of their decreased purification capacity; pollution caused by fossil fuels, their limited life span, and increased prices. Mono alkyl ester biodiesel is a clean diesel fuel that can be manufactured by fresh or used vegetable oils or even animal fats and waste oils. This fuel is made from renewable sources and is biodegradable [1–3]. Transesterification method or green process is one way of producing biodiesels. In this method, vegetable oils are added to an alcohol (usually methanol) producing esters. It is noteworthy that glycerol is the byproduct of this reaction which greatly contributes to the pharmaceutical science and cosmetic products [4].

Parameters affecting transesterification procedure are: reaction temperature, alcohol-oil mole proportion, humidity, free fatty acids, mixing speed, alcohol type, reaction duration, catalyst type and amount [5–7]. Catalysts used in transesterification procedure are categorized into 3 groups: homogenic catalysts, heterogenic catalysts, and biocatalysts [8, 9]. The limitations of homogenic catalysts have resulted in the dominant use of the heterogenic catalysts. Heterogenic catalysts and reactants are in separate phases and are divided into acidic and basic solid catalysts. ZrO and TiO are examples of acidic solid catalysts, while MgO and CaO are among the basic solid ones [10, 11]. It is worthy to mention that basic solid catalysts happen to be more effective than the acidic ones because they do not require high temperature and pressure, thus react more rapidly, have fewer side reactions, have no oxidation, and are more available and cost-effective [12, 13]. Heterogenic catalysts are beneficial in many ways: a) They decrease soaping problems and water usage, b) they do not mix with methanol or ethanol, c) they are easy to separate from biodiesel and glycerol, d) they are recyclable and can be reused many times, e) they are environmentally compatible and can be used in continuous and non-continuous procedures, f) they have low sensitivity to free fatty acids because of the stability of their surface microcrystal structure, and g) their quality of biodiesel is high, although they convert triglyceride to biodiesel rather slowly [14, 15].

In fact, the most important problem in producing biodiesel is its high cost which depending on the primal raw material can be 1.5 times the prices of petroleum diesel. Reports? almost 80 % of the total biodiesel cost comes from primal materials cost which are vegetable oils or animal fats. Thus, using burnt food oil decreases the related costs in producing biodiesel because of its availability and inexpensiveness. In addition, environmental pollution is lessened by recycling used cooking oils [16–18]. Used cooking oils have characteristics that make them different rather than vegetable oils or animal fats. High cooking temperature and food water hydrolyze triglycerides produce lots of free fatty acids. Reaction between these free fatty acids and water can form soaps and reduce biodiesel efficacy. Free fatty acids make up 0.5–15 % of used cooking oil weight, although the proper amount for transesterification reaction is less than 0.5 % of weight percentage [19, 20].

Basic heterogenic catalysts are divided into metallic oxides and their derivatives. CaO is one type of basic heterogenic catalysts which is most widely used for transesterification reaction because it is highly basic, and has a low solubility in methanol; it can be synthesized from inexpensive sources such as lime stone and calcium hydroxide and generates low bioenvironmental pollution. Nanocatalysts have high specific surface and degeneration activity. Other studies have shown that high specific surface and large porosity is beneficial for the catalyst to bond with the sub-layer which enhances the efficacy of transesterification reaction. One way to produce Nano CaO is to saturate lithium on CaO through wet method. It is suggested that a 12:1 proportion of methanol to oil at 65 C heat in 2 h using 5 % weight of the catalyst for esterification of jatropha oil has a yield over 99 % [21].

The first basic heterogenic catalyst in pilot scale used for transesterification reaction was MgO [18]. Other studies have indicated that MgO has a fine efficacy in non-continuous reactions. Production costs of biodiesel products in a discontinuous reactor decrease in the presence of MgO. MgO has a high yield in highly critical conditions and in a high proportion of methanol to oil [11, 22]. The following results are achieved in other studies concerning MgO catalyst (Table 1) [10].

**Table 1:** The efficacy of methyl ester using MgO as catalyst

Type of Oil	Surface Area (M^2^/g)	Reaction’s Temperature (^o^ C)	Molar ratio Methanol/oil	Reaction’s Duration (hour)	Catalyst concentration (% W/W)	Efficiency
Linseed Oil	300	Methanol-Reflux	75:1	22	10 %	64 %
Soybean Oil	36	180	12:1	1	5 %	72 %
Soybean Oil	229	180	12:1	1	5 %	90 %

Nano MgO exhibits a high yield for soy oil transesterification reaction in the presence of super critical methanol. This catalyst’s solubility and penetration plays an essential role in enhancing the reaction efficacy, which also can be adjusted by mixing two catalysts. For instance, 90 % yield is achieved by soy oil in a 10-min reaction, using 36:1 methanol to oil ratio, 533 K temperature, 3 % weight catalyst, and 1000 rpm [23, 24].

Another tranesterification reaction was performed on cotton seed oil, in the presence of 14.4 % weight CaO-MgO catalyst, loaded on Al2O3, using 12:24 methanol to oil ratio, 95.63 °C temperature, and 97.62 % yield was achieved. Two types of commercial nanopowder calcium oxides were studied in order to discuss the transesterification reaction of canola oil: Nanopowder exhibiting a higher surface area (HSA nano-CaO) and nanopowder exhibiting a moderate surface area (nano-CaO). The effects of reaction temperature, catalyst/oil weight ratio, and methanol/oil molar ratio on the reaction’s conditions were studied. The results claim that nanopowder CaOs present a higher activity than the other type, due to their larger BET surface areas. At 65 °C, 99.85 % biodiesel yield was obtained by 3 wt. % of the nano-CaO catalyst, and 9:1 methanol/oil molar ratio, in 2 h. The required catalyst/oil weight ratio in order to achieve the same yield under the same conditions, was 10 times less in the HSA-nano-CaO catalyst’s case [25].

In this study, Nano CaO and Nano MgO were synthesized, using sedimentation method and sol-gel self-combustion, respectively. While the next step was progressing, transesterification reaction was performed and examined in different ways with various proportions of catalyst weight and mole proportion of alcohol to oil. Also, catalyst repeatability was assessed. This study was aimed at comparing the efficacy of the reaction with and without catalysts by coupling Nano CaO and Nano MgO. Using a combination of 2 catalysts (MgO and CaO) results in an enhanced basic strength and contact surface. Subsequently, the reaction is performed more gently and is more repeatable.

## Experimental

### Materials

Analytical reagent (AR) grade calcium nitrate, ammonia, urea, sodium hydroxide, potassium hydroxide, magnesium sulfate, methanol, 2-propanol, and phenolphthalein were purchased from Merck. Co. Ethylene glycol and magnesium nitrate used in this study were obtained from Aldrich. Co. Moreover, home waste cooking oil was also provided. All other chemicals were obtained commercially and of analytical grade No additional purification was done on materials.

### Apparatus

Oven temperature programmed (up to 1200 C), X-ray diffraction (XRD) (STADI-P, STOE, Germany), scanning electron microscope (SEM) (Kyky 3200, China), field emission scanning electron microscope (FESEM) (S-4160, Hitachi, Japan), IKA-WERKE-EUROSTAR digital mechanical mixer (Germany, 50–2000 rpm), and Fourier transform infrared spectrometer (FTIR) (BRUKER, TENSOR2, Germany) were provided for conducting the study.

### Catalyst preparation

#### Nano CaO synthesis using sol-gel method

Firstly, 11.81 grams calcium nitrate.4H_2_O was dissolved in minimum amount of water and then it was added to 25 ml of ethylene glycol being mixed by mechanical mixer. Two grams of NaOH was dissolved in 25 ml of purified water, added drop by drop, as the mixing took place. The produced mixture was mixed for 2 h so that a clear white gel was obtained. The white gel was kept still for 2 h for the reaction to complete. Then after 4 times of being washed with water, NaOH was excreted and pH was adjusted at about 10. After that, the gel was heated up to 80 °C for 2 h so that the water could be vaporized and the gel concentrated. The concentrated gel was placed in a desiccator for one hour to dry completely. The resulting dry gel was milled in the form of a white powder. The produced powder was put in the cruse and into the oven which was gradually heated up to 800 °C and stayed so for an hour for de-carbonization.

#### Nano MgO synthesis using sol-gel self-combustion with urea fuel

Initially, 21.58 g of magnesium nitrate.6H_2_O and 10.16 g of urea were dissolved in non-ionized water at the mole ration of 1:2. Ammonia was added drop by drop till pH of the mixture was 7. The system was placed in a 60–70 °C water bath and stirred by a mixer to allow the water and mobile materials present in the mixture to evaporate. As water vaporized, the solution was concentrated and became viscose. The resulting gel underwent self-combustion; as a result, it started boiling and surfaced the walls of the crucible. Subsequently, a thermo genic reaction took place and a lateritious gas was emitted. This reaction left a white porous mass. NO_3_^−^ ions in the compound created an oxidative environment for the degradation of organic mixtures. In order to eliminate the remaining organic material, the powder from self-combustion reaction must be de-carbonized at higher temperatures. Next, the powder was fined and put in a furnace (600 °C for 2 h) to de-carbonize.

### Catalyst characterization

To investigate the structure and crystallinity of the catalysts, the X-ray diffraction analysis was conducted. Morphology and the mean particle size of the catalysts were analyzed by SEM and XRD spectrums. In addition, the microstructure was characterized by field emission scanning electron microscopy.

### Transesterification reaction of cooking waste oil using methanol in presence of synthetized Nano CaO

The yield for waste cooking oil transesterification reaction in the presence of Nano CaO catalyst has been examined according to previous studies [16, 17].

Overall, 40 g of waste oil was filtered using Buchner funnel in vacuity and then 0.5 g MgSO4 was added while mixing to trap the Water mixed with oil. After filtering the waterless oil was poured into a 100 ml flask using stirrer and it was heated to 55 °C for 20 min. Then 0.4, 0.6, and 1.2 g of the catalyst and 8.76, 10.22, and 11.69 g of methanol were added to it. The heater was warmed to 75 °C so that methanol reflux was engaged. After 4 and 6 h, the mixture was moved to a decanter and over time 2 phases were shaped.

The final products of this reaction are biodiesel and combined glycerin with Nano CaO.

The upper phase was biodiesel and the lower was combined glycerin with Nano CaO.

**Fig. 1:**
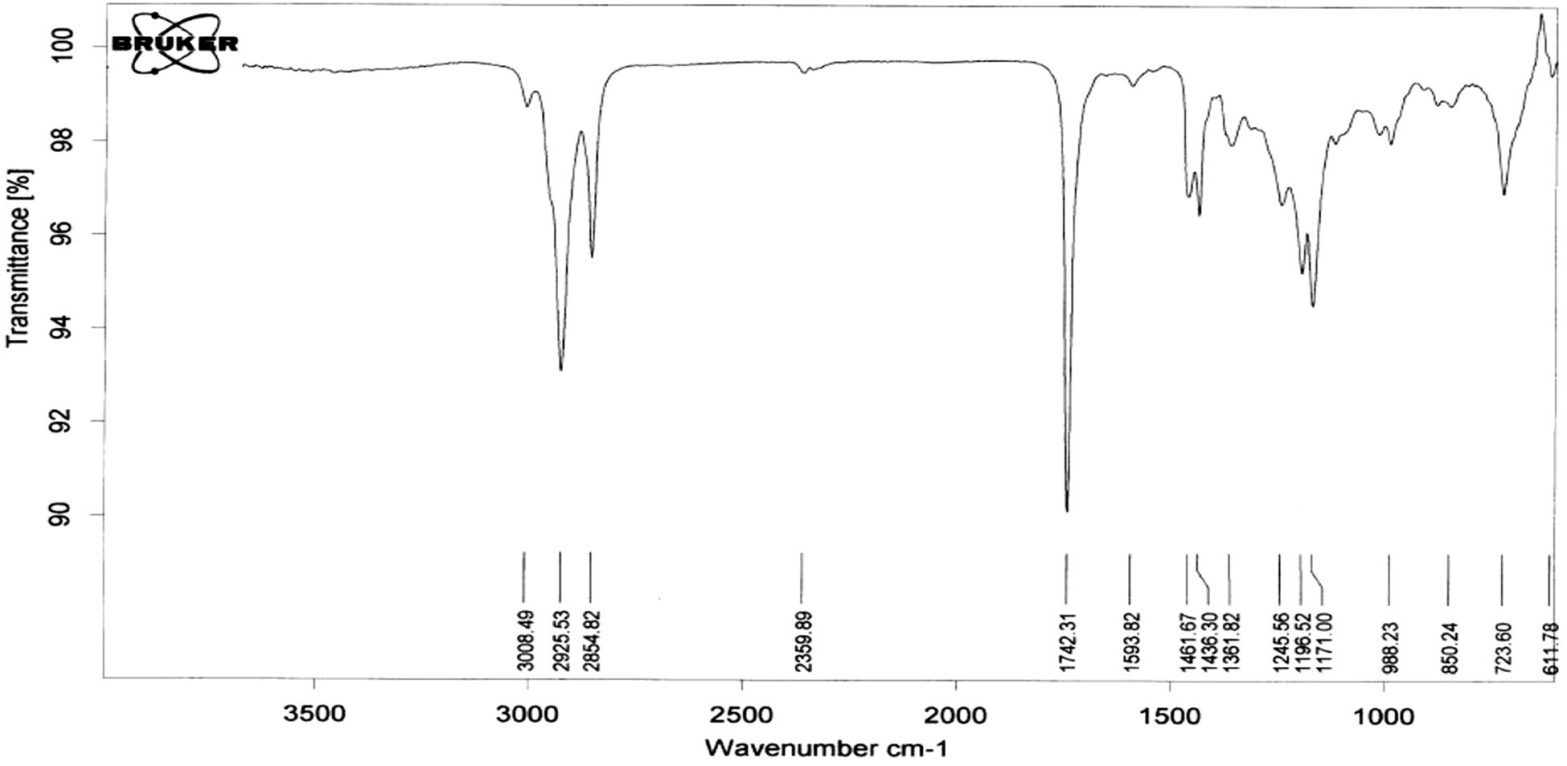
Characteristics of synthetic Nano CaO: (**a**) SEM image; (**b**) XRD spectrum

The phases were separated and the biodiesel was returned to the decanter for washing. Then, 40mls of the 80° water was added to the biodiesel so that its pH reached 7. Moreover, the water used in this process was clear and neutral after the first washing. The produced biodiesel had some amount of water and magnesium sulfate was used to absorb it. Then the waterless biodiesel was separated from MgSO_4_ by centrifuge. In order to check the purity, IR spectrums of biodiesel and oil were compared (Fig. 1).

#### The tranesterfication reaction of cooking waste oil using methanol in the presence of synthetized Nano MgO via self-combustion method

The yield for waste cooking oil transesterification reaction in the presence of Nano MgO catalyst using self-combustion was studied with alcohol-oil proportions of 1:30, 1:20, 1:12, 1:7, and 1:6 and 1.5, 3, 5 and 10 % weight catalyst during 6 and 12 h. It should be noted that yield was not sufficient in any of these conditions. Ten grams of waste oil was filtered using Buchner funnel in vacuity and then 0.5 g of magnesium sulfate was added while mixing to trap the oil water. After filtering the waterless oil was poured in a 100 ml flask with a magnet, it was heated to 55 °C for 20 min. Then 0,0.5, 1, 0.5, and 3.1 g of the catalyst and 2.2, 4.28, 10.7, 14.61, 55.19, and 95.4 g of methanol were added to it. The heater was warmed to 75 °C so that methanol reflux was engaged. After 12 h, the mixture was moved to the decanter but only a single yellow oily phase was formed under which was the catalyst.

It showed that transesterification reaction hadn’t made any progress significantly in the presence of the catalyst.

#### The trasnesterification reaction of cooking waste oil using methanol in the presence of a combination of Nano CaO and MgO prepared by self-combustion method

The protocol for preparing oil was the same as what was explained earlier in this article only with the difference that here, 3 % weight of self-combustion combination of the catalysts, MgO and CaO, and 7:1 oil to alcohol proportion were used. The 3 % weight meant 1.2 g of the catalysts mixture which was generated by changing the mass proportion of the two catalysts of the transesterification reaction.

### Recycling of catalyst

#### Recycling Nano CaO catalyst and evaluating its repeatability in waste cooking oil transesterification reaction

After transesterification reaction and separation of the biodiesel from glycerol and the catalyst, the remaining catalyst was washed away with n-Hexane and placed in a 70° oven for 2 h to dry. In the next step oil preparation and the reaction procedure were done as described. The catalyst could be used up to six times.

#### Recycling the catalysts Nano CaO (0.7 g) and self-combustion Nano MgO (0.5 g) and their repeatability in waste cooking oil transesterification

After transesterification reaction and separation of the biodiesel from glycerol and the catalyst, the remaining catalyst was washed away with n-hexane and it was placed in a 70° oven for 2 h to dry. In the next step, oil preparation and the reaction procedure were done as described earlier in this article. The catalyst mixture presented a repeatability of up to eight times.

## Results

### Catalyst characterization

**Fig. 2:**
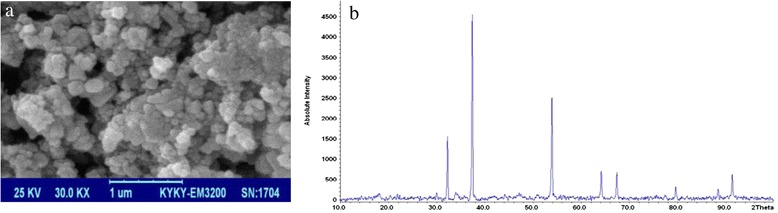
Characteristics of synthetic Nano MgO: (**a**) SEM image; (**b**) XRD spectrum

**Fig. 3:**
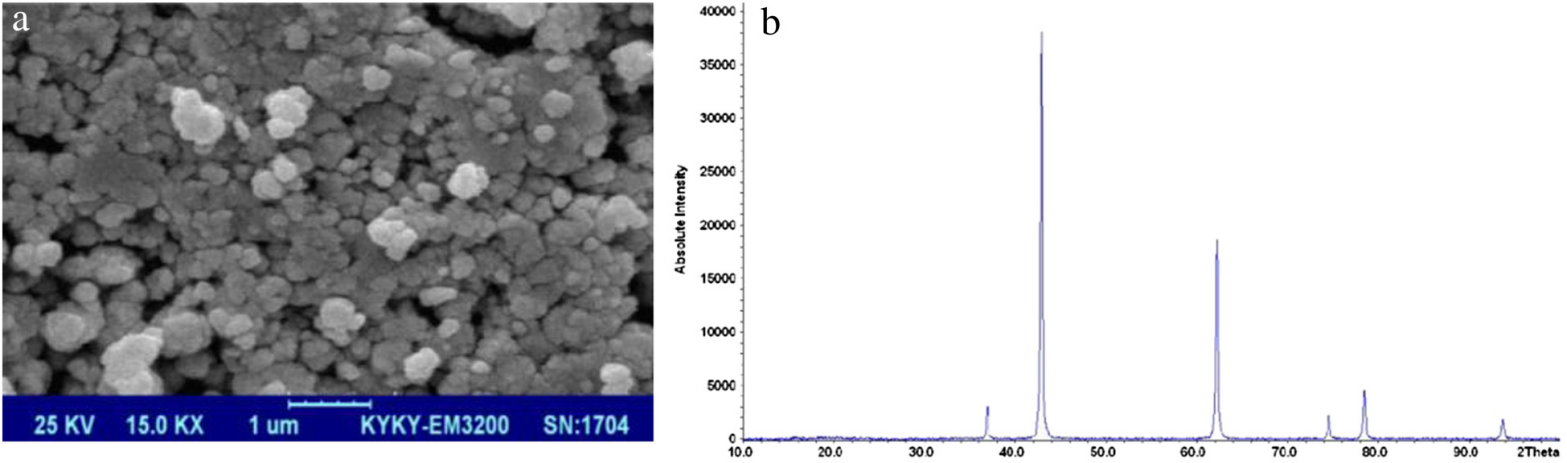
Biodiesel FTIR spectrum in the presence of Nano CaO

SEM images indicated a spherical shape for the Nanoparticles (NPs) and CaO and MgO NPs were determined to be 65 and 70 nm in size. The application of XRD showed that the crystal structure of the particles was cubic. Additionally, the comparison of the obtained peaks and source peaks indicated that the Cao NPs were synthesized and average particle size was calculated 61 nm according to Scherer’s equation (Fig. 2a, b). Also, the comparison of the obtained peaks and source peaks indicated that the Mgo NPs were synthesized and average particle size was calculated 69 nm according to Scherer’s equation (Fig. 3a, b).

### Effect of Catalyst Compound on biodiesel yield

#### Waste cooking oil transesterification reaction yield in the presence of synthesized Nano CaO catalyst

As described earlier in Section 2.5.1, this reaction was performed using different proportions of methanol to oil and various catalyst weight percentages. The results showed that 1:7 mol proportion for alcohol to oil and 1.5 % weight catalyst over 6 h are the optimum conditions for this reaction. Biodiesel production mass yield is 94.37 % in these optimum conditions (Table 3).

#### Waste cooking oil transesterification reaction yield in the presence of synthesized Nano MgO catalyst

Following the methods described in Section 2.5.2, this reaction was performed using different proportions of methanol to oil and various catalyst weight percentages. Nevertheless, transesterification reaction did not significantly progress in the presence of this catalyst.

#### Waste cooking oil transesterification reaction yield in the presence of 3 % combined Nano CaO and self-combustion Nano MgO catalysts and alcohol to oil mole proportion of 1:7

Waste cooking oil transesterification reaction yield in the presence of combined oxide catalysts was studied and it indicated that by combining Nano CaO and self-combustion Nano MgO with the 3 % weight percentage and alcohol to oil mole proportion of 1:7 over 6 hours, biodiesel production mass yield rose by increasing CaO mass proportion to MgO. These studies show that although self-combustion Nano MgO does not help the transesterification reaction while combined with Nano CaO, it increases the biodiesel production mass yield. The optimum mass proportion for CaO to MgO is 0.7:0.5 that gives a mass yield of 98.95 %. Results given in Table 3 show the progressive biodiesel production mass yield increased with Nano CaO to self-combustion Nano MgO with mass proportion change (Table 5).

### Recycling of catalyst

#### Waste cooking oil transesterification reaction with Nano CaO repeatability

**Fig. 4:**
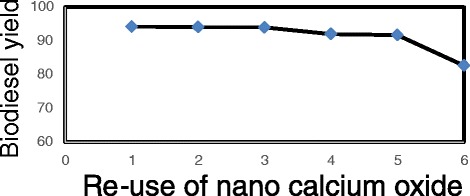
The efficacy of biodiesel production in six repetitions of Nano CaO

After transesterification reaction in the presence of CaO the catalyst was separated from the main products and washed away with n-hexane and put in a 70° oven to dry. Then in optimum conditions of the reaction, repeatability was evaluated. The results and the number of repetitions are shown in Fig. 4.

#### Waste cooking oil transesterification reaction repeatability with Nano CaO and Nano MgO catalyst by the proportion of 0.7–0.5 g

**Fig. 5:**
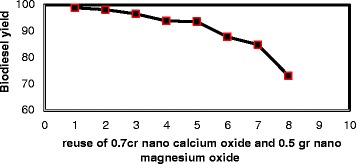
The efficacy of biodiesel production in eight repetitions of CaO (0.7g)+MgO (0.5g) Nanocatalysts

When the optimum mass proportion for CaO to self-combustion MgO was determined and transesterification was over, the combination of the catalysts was separated from the main product and washed with n-hexane, then it was placed in a 70° oven to dry. Eventually, repeatability was determined for the catalyst mixture in optimum conditions. The results and number of repetitions are shown in Fig. 5.

### FTIR analysis

IR spectrum for methyl esters (biodiesel) is somewhat similar to oil spectrum and it rarely has some differences in the finger print area. As the only change in oil structure is the exit of glycerol and substituting methanol in hydrocarbon chain, methyl ester IR spectrum is much like triglyceride oils and the most variations are in 1000–1500 area by which can be verified by the complete synthesis of the biodiesel. The three main effective groups are shown in Table 2.

**Table 2:** The three main agent groups of biodiesel

Frequency (cm ^−1^)	The Functional Group	Type of Vibration	Intensity
1436	-O-CH_3_	Bending	Moderate
1196.52	-O-CH_3_	Rocking	Moderate
1171	-C-O-(CH_3_)	Stretching motions symmetric	High

**Fig. 6:**
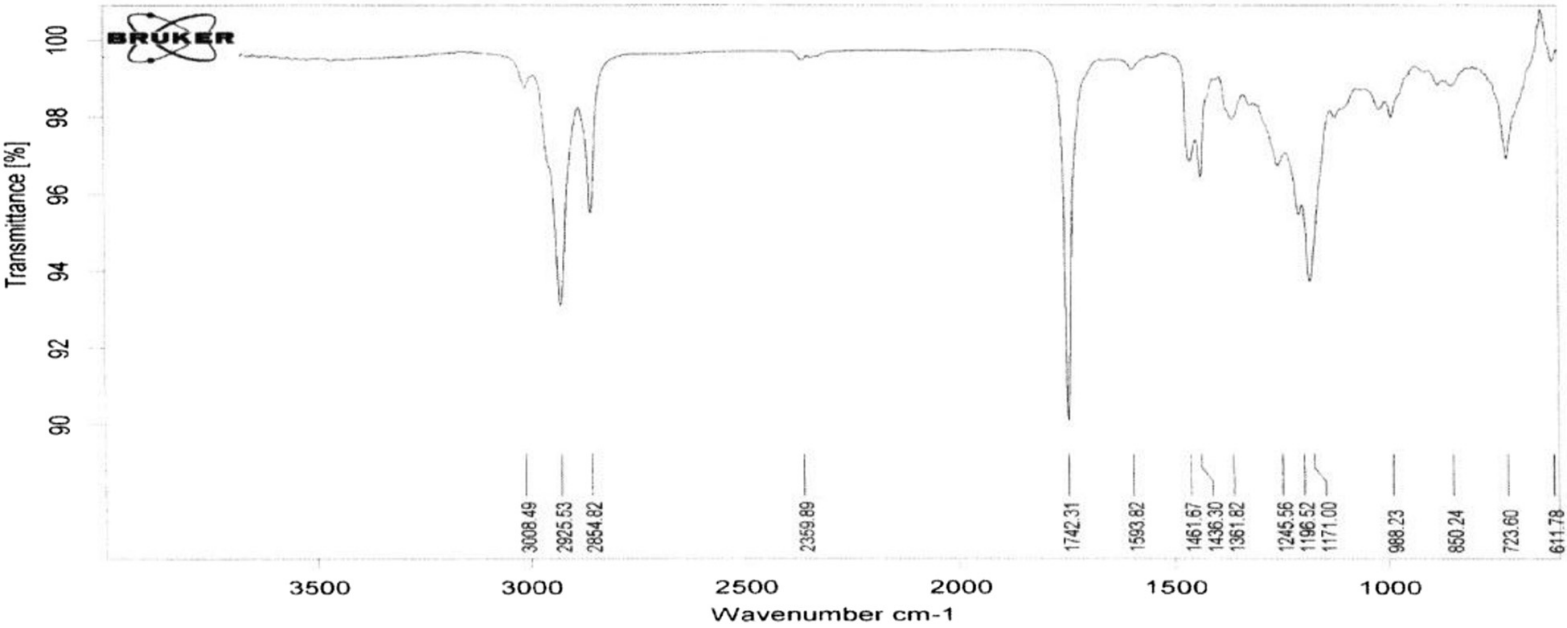
Biodiesel FTIR spectrum in the presence of 0.7g of Nano CaO and 0.5g of Nano MgO

In addition, the lack of -OH spectrum near 3500 region implies that the transesterification reaction is performed completely and lack of intermediate compounds like free fatty acids and mono and diglycerids can be also rationalized (Fig. 5). The resulting spectrum showed that transesterification did not make any progress much in the presence of Mgo Nanocatalyst and the three main effective groups were not recognizable in the spectrum. Additionally, transesterification in the presence of catalyst mixture progressed completely and the three main effective groups emerged in the spectrum (Fig. 6).

## Discussion

During the first step of tranesterification reaction in the presence of Nano CaO, proton is absorbed from methanol by means of basic sections available in Nano CaO structure. This reaction results in meth oxide anion formation. Meth oxide group holds an oxygen molecule within its structure which attacks carbon from triglyceride-fatty acid‘s carbonyl group, resulting in alkoxycarbonyl formation as an intermediate structure. Alkoxycarbonyl transfroms itself into more stable products: fatty acid methyl ester and diglyceride. As the transformation continues, diglyceride converts to monoglyceride and fatty acid methyl ester. During the third step, monoglyceride undergoes the same reaction and produces glycerin and fatty acid methyl ester. In conclusion, fatty acid tri-methyl ester and glycerin are the final products of the reaction. The summarized version of reaction is provided below:

R1,R2,R3 = fatty acid carbonic chain

Alcohol’s alkali group = R4

Nano CaO provides us with a better efficacy and using waste cooking oil is more cost-effective [16]. Optimum conditions for this catalyst is 1:7 methanol to oil, in six hours and 1.5 % of catalyst’s weight at which biodiesel production yield is 94.37 % of weight (Table 3).

**Table 3:** The efficacy of biodiesel production in the presence of synthetic Nano CaO catalyst

The Amount of Oil (gr)	The Catalyst Compound	Alcohol: Oil Mole Ratio	Mass Percent of the Catalyst Compound	Reaction’s Duration (hr)	Mass Efficiency
40	CaO	6:1	1 %	4	78.35
40	CaO	6:1	1.5 %	4	80.12
40	CaO	6:1	3 %	4	80.18
40	CaO	7:1	1.5 %	4	92.39
40	CaO	7:1	3 %	4	92.33
40	CaO	8:1	1.5 %	4	92.36
40	CaO	7:1	1.5 %	6	94.37

In 2008, Kouzu M. released an article, concerning Calcium oxide as a solid base catalyst for transesterification of soybean oil and its contribution to biodiesel production.

Soy fatty acid methyl ester production yield in the presence of CaO is reported 93 % in one-hour time duration and methanol-oil mole proportion of 12:1 [26, 27]. In 2010, Man kee Lam provided an article, concerning Homogeneous, heterogeneous and enzymatic catalysis for transesterification of hight free fatty acid oil (waste cooking oil) to biodiesel.

It was observed that when using 0.85 g CaO and under same conditions, yield decreases to 66 % [28]. Results obtained from different studies for CaO indicate that (Table 4):

**Table 4:** The efficacy of methyl ester using CaO as catalyst for 15 min

Type of Oil	Reaction’s Temperature (^o^ C)	Molar ratio Methanol/oil	Catalyst concentration (% W/W)	Efficiency
Linseed Oil	Methanol-Reflux	4.5 : 1	0.8	10 %
Linseed Oil	192	41.1 : 1	3	50 %

By comparing obtained results from this survey, and other conducted surveys, it can be concluded that Nano CaO can enhance trans esterification reaction’s efficacy more than CaO owing to its high contact area. Repeatability of Nano CaO and the ability of reusing this catalyst were also discussed. As the results indicate, repeatability of this catalyst was satisfactory (Fig. 4).

Nano MgO is not catalyzing the transesterification by itself, but when used with Nano CaO transesterification reaction yield significantly increases. By enhancing MgO’s catalytic strength and basic strength, the reaction is performed in more gentle conditions (Table 5).

**Table 5:** The efficacy of biodiesel production in the presence of CaO + MgO Nanocatalyst

The Amount of Oil (gr)	The Catalyst Compound	Alcohol: Oil Mole Ratio	Mass Percent of the Catalyst Compound	Reaction’s Duration (hr)	Mass Efficiency
40	CaO + MgO	7:1	0.5 g CaO+ 0.7 g MgO = 3 %	6	90.85
40	CaO + MgO	7:1	0.6 g CaO+ 0.6 g MgO = 3 %	6	96.22
40	CaO + MgO	7:1	0.7 g CaO+ 0.5 g MgO = 3 %	6	98.95
40	CaO + MgO	7:1	0.9 g CaO+ 0.3 g MgO = 3 %	6	98.57

The comparison of the obtained values with the standard tests indicates that they all fall within the normal range and as a result, this biodiesel is viable (Table 6).

**Table 6:** Standard test results for biodiesel were produced from waste cooking oil in the presence of combined Nano CaO and self-combustion Nano MgO catalyst 0.7:0.5 compared to D675-01 ASTM and EN14217

Property	The Standard Value	The Permissive Range	The Measured Value
Flash Point	D93	Minimum Value:130	150 (^o^ C)
Kinematic Viscosity in 40^o^ C	D445	1.6–9	4.8 mm^2^/s
Cetane Number	D613	Minimum Value:47	48
Saponification Number	D460	Indefinite	190.70
Freezing Point	-	-	−7 (^o^ C)
Ignition Point	-	-	220 (^o^ C)
Cloud Point	D2500	Indefinite	4 (^o^ C)
Pour Point	ISO3016	Maximum Value:0	−5 (^o^ C)
Iodine Number	EN14111	Maximum Value:130	124.48
Density	EN ISO 3675	0.86–0.89 (gr/cm^3^ )	0.89 (gr/cm^3^ )

## Conclusions

As the results indicate, Nano CaO provides us with a better efficacy, reaction duration, repeatability, used catalyst weight percentage, methanol amount, and biodiesel production mass yield in comparison with self-combustion Nano MgO, due to its basic nature. Nano MgO is not capable of catalyzing the transesterification by itself, because it has a much weaker basic affinity but when used with Nano CaO due to its surface structure, the basic properties increase and it becomes a proper base for the catalyst so that CaO contact surface increases and transesterification reaction yield significantly increases as well. Needless to say that higher proportions of Nano CaO to Nano MgO lead to increase in biodiesel production mass yield. The optimum condition for this catalyst is 1:7 methanol to oil (the same ratio which is applied for Nano CaO), 6 h and 0.7 g of Nano CaO plus 0.5 g of Nano MgO which gives the yield of 98.95 % of weight. Moreover, combined catalyst repeatability is better than Nano CaO alone. In addition, using waste cooking oil is more cost-effective.
